# Gamma-delta (γδ) T cells: friend or foe in cancer development?

**DOI:** 10.1186/s12967-017-1378-2

**Published:** 2018-01-10

**Authors:** Yijing Zhao, Chao Niu, Jiuwei Cui

**Affiliations:** Cancer Center, The First Hospital of Jilin University, Changchun, 130021 People’s Republic of China

**Keywords:** γδ T cells, Adoptive immunotherapy, Protumor, Antitumor, Tumor microenvironment, Cytokine, Polarization

## Abstract

**Background:**

γδ T cells are a distinct subgroup of T cells containing T cell receptors (TCRs) γ and TCR δ chains with diverse structural and functional heterogeneity. As a bridge between the innate and adaptive immune systems, γδ T cells participate in various immune responses during cancer progression. Because of their direct/indirect antitumor cytotoxicity and strong cytokine production ability, the use of γδ T cells in cancer immunotherapy has received a lot of attention over the past decade.

**Main text:**

Despite the promising potential of γδ T cells, the efficacy of γδ T cell immunotherapy is limited, with an average response ratio of only 21%. In addition, research over the past 2 years has shown that γδ T cells could also promote cancer progression by inhibiting antitumor responses, and enhancing cancer angiogenesis. As a result, γδ T cells have a dual effect and can therefore be considered as being both “friends” and “foes” of cancer. In order to solve the sub-optimal efficiency problem of γδ T cell immunotherapy, we review recent observations regarding the antitumor and protumor activities of major structural and functional subsets of human γδ T cells, describing how these subsets are activated and polarized, and how these events relate to subsequent effects in cancer immunity. A mixture of both antitumor or protumor γδ T cells used in adoptive immunotherapy, coupled with the fact that γδ T cells can be polarized from antitumor cells to protumor cells appear to be the likely reasons for the mild efficacy seen with γδ T cells.

**Conclusion:**

The future holds the promise of depleting the specific protumor γδ T cell subgroup before therapy, choosing multi-immunocyte adoptive therapy, modifying the cytokine balance in the cancer microenvironment, and using a combination of γδ T cells adoptive immunotherapy with immune checkpoint inhibitors.

## Background

γδ T cells are a subgroup of T cells with distinct T cell receptors (TCRs) γ and δ chains on their surface, which account for 0.5–5% of all T-lymphocytes. This small subset of cells were first found in 1987, after the accidental discovery of third chain of the TCR (γ chain) in 1984 [1–3]. In contrast, the most T cells in normal human body are αβ T cells (65–70%) with TCR composed of two glycoprotein chains called α and β TCR chains. These cells are generally simply referred to as “T cells”. Although, γδ T cells are much less common than αβ T cells, they are at their highest abundance in the gut mucosa, within a population of lymphocytes known as intraepithelial lymphocytes [3, 4]. As outstanding research on γδ T cells ever since, these immune cells have gained close attention than ever before. Their features include non-MHC restricted antigen recognition and an abundant cytokine secretion capacity, suggesting that they possess a high antitumor capability. These attractive features have raised expectations for their application in cancer adoptive immunotherapy [5]. Up until now, clinical trials have been conducted in numerous cancers, such as renal cell carcinoma, malignant leukemia, and advanced lung cancer, as well as others, with the majority of trials showing them to be well tolerated and safe [4–7].

However, in recent years, there have been a number of ongoing reports claiming that γδ T cells promote cancer development (i.e. have protumor activity) [8, 9]. For example, γδ T17 cells are one of the major sources of IL-17 in the cancer microenvironment [10], and IL-17 can promote cancer growth by supporting angiogenesis in gall-bladder cancer, gastric cancer, non-small cell lung carcinoma, as well as other cancers [11–14]. It has also been reported that γδ T cells can increase the population of myeloid derived suppressor cells (MDSCs). MDSCs have been reported to facilitate cancer progression in several types of cancer, such as esophageal cancer, breast cancer, colorectal cancer and pre-hepatic carcinoma [15–19].

A review of clinical trials conducted in the last decade shows that γδ T cell-based immunotherapies are safe and well tolerated. However, the clinical benefits appeared to be mild to moderate at best and raise a number of questions. Can γδ T cells inhibit cancer growth on the one hand, and promote cancer development on the other? What controls the efficiency of γδ T cell-based cancer immunotherapy? This review will uncover the mystery of the dual effects of γδ T cells in cancer immunotherapy. From “foe” to “friend,” we turn our attention away from their well-known immune effector role and toward to their new-found immune suppressive regulatory role. By carefully reviewing the last decade of clinical trials and pre-clinical research, we suggest that the limited efficacy of γδ T cell therapies may be caused by the different effects of specific γδ T cells subgroups on cancer cells, as a result of their polarization following cytokine stimulation. Based on these finding, we propose that future immunotherapeutic should focus on promoting antitumor γδ T cell proliferation, while at the same time suppressing protumor γδ T cells. Additionally, breaking the suppressive tumor microenvironment (TME) will be another means of improving the antitumor efficiency of γδ T cells. In particular, the most promising strategy for future γδ T cell immunotherapy is proposed to be modification of the cytokine balance in the TME coupled with deletion of the specific protumor γδ T cell subgroup.

## Classification of γδ T cells

**Fig. 1:**
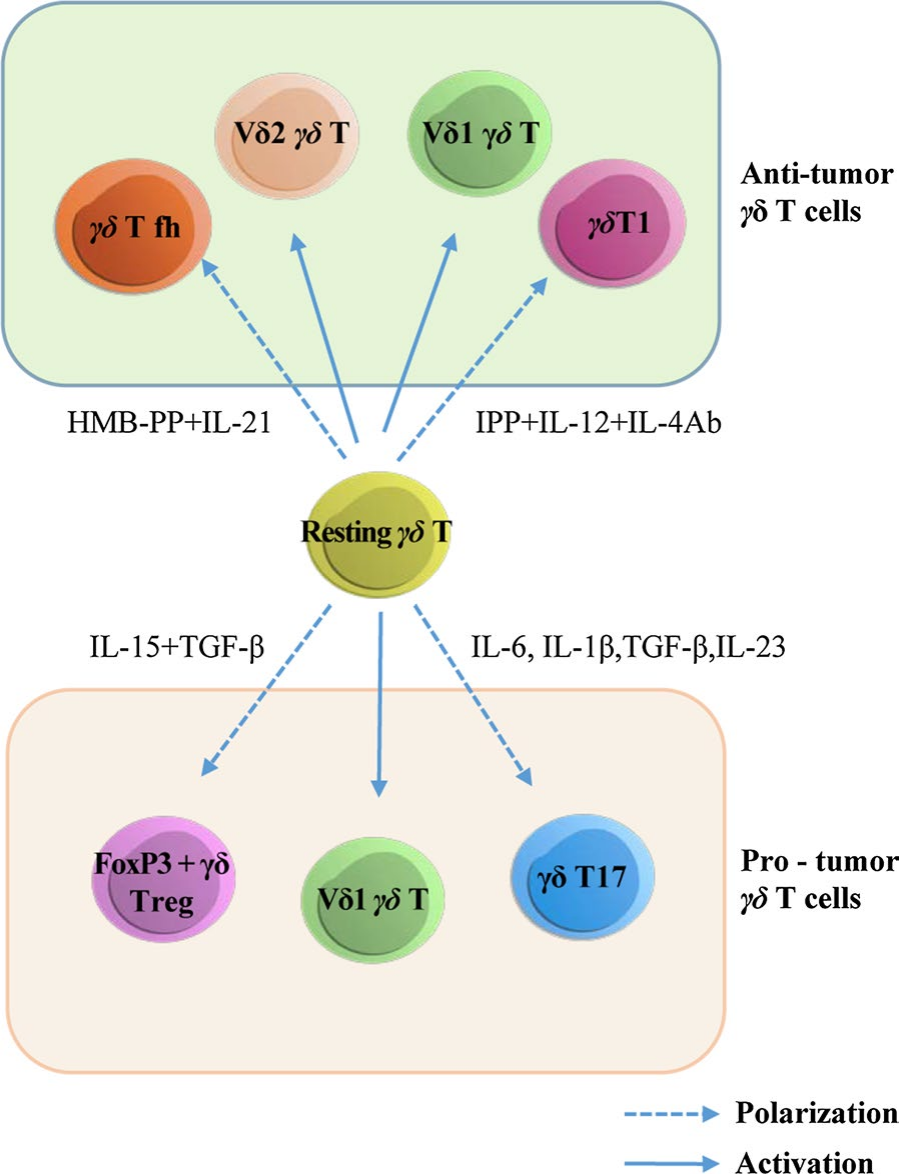
Classification of γδ T cells towards tumors. Vδ1 γδ T cells and Vδ2 γδ T cells are normal resting γδ T cells before activation or polarization. Following stimulation with different cytokines, resting γδ T cells can polarize into the protumor subgroups: FoxP3+ γδ Treg, γδ T17, or become activated Vδ1 γδ T cells. In addition, resting γδ T cells can also polarize into the antitumor subgroup: γδ Tfh, and γδ T1, or become activated Vδ1 γδ T cells

γδ T cells are a group of heterogeneous T cells, composed of a variety of subgroups, based on their TCRs composition and cellular function. The combinatorial variety generated by the different TCRs are thought to underlie the reason γδ T cells exert diverse actions in distinct pathological types of cancer (Fig. 1). As the name suggests, the γδ T cell receptor contains δ and γ chains. Based on the TCR structure, human γδ T cells can be divided into four main populations based on TCR δ chain expression (δ1, δ2, δ3, δ5) [20, 21]. Furthermore, the different TCR δ chains and TCR γ chains combined together to form different γδ T cell types [22, 23] (Table 1). For example, γδ T cells expressing a TCR containing γ-chain variable region 9 (Vγ9) and δ-chain variable region 2 (Vδ2), are referred to as Vγ9 Vδ2 T cells, and these cells represent the majority of γδ T cells in peripheral blood [24]. In both humans and mice, Vγ2, Vγ3, Vγ4, Vγ5, Vγ8, Vγ9, and Vγ11 rearrangements of the γ chain are found; in addition several Vγ pseudo-genes (Vγ1, Vγ5P, Vγ6, Vγ7, and Vγ10) are present in mice but not in humans [23]. As has been shown in numerous pre-clinical and clinical studies, Vγ9 Vδ2 T cells have potent antitumor activity. They can inhibit cancer cell proliferation, angiogenesis, lymphangiogenesis, and increase cancer cell apoptosis [25]. Vγ9 Vδ2 T cells can recognize phosphorylated antigens that accumulate in cancer cells, interact with the F1-ATPase expressed at the cancer cell surface, and recognize stress-induced molecules, such as the MHC class I chain-related molecules A and B (MICA and MICB), as well as UL16-binding proteins [26]. In contrast, Vδ1 and Vδ3 γδ T cells comprise only a minor subset of T lymphocytes. Vδ1 γδ T cells are found in normal human epithelia, dermis, spleen, and liver, as well as being found in the peripheral blood of patients with chronic viral infections and patients with leukemia [27]. Vδ1 γδ T—cells expanded from peripheral blood exhibit a specific cytotoxicity against B cell chronic lymphocytic leukemia-derived cells [28]. Vδ3 γδ T cells are found in the liver and the gut epithelium [29, 30], which is rarely studied in cancer. Compared with Vγ9 Vδ2 T cells, tumor-reactive Vδ1 γδ T cells do not preferentially pair with a specific Vγ chain, and they can persist in circulation for a long time after stimulation [31]. Nevertheless, their role in cancer is controversial, and this subgroup will be discussed further in a later section.

**Table 1:** Structural subsets of human γδT cells

Structure subset	Paired Vγ gene	Distribution
Vδ1	Vγ2, Vγ3, Vγ4, Vγ5, Vγ8, Vγ9	PB, skin, gut, spleen, liver
Vδ2	Vγ9	PB
Vδ3	Vγ2, Vγ3	PB, liver
Vδ5	Vγ4	PB

Based on their function, γδ T cells can be divided into two subsets: effector γδ T cells and regulatory γδ T cells. The regulatory and effector functions of γδ T cells have recently been excellently reviewed by Paul and Lal [34]. When γδ T cells are activated by a stimulus, these cells, which play an antitumor role by secreting cytokines, act through antibody dependent cellular cytotoxicity (ADCC) effects, as well as other processes, are referred to as effector γδ T cells. In contrast, γδ T cells, which are responsible for modulating the immune system and maintenance of immunological tolerance, are referred to as regulatory γδ T cells (γδ Treg cells), and this subgroup is also named as γδ suppressor T cells [32–34]. γδ Treg cells promote cancer growth by impairing the function of various effector cells. γδ Treg cells can induce immuno-senescence by targeting naïve T cells, as well as dendritic cells (DCs) [35]. γδ T17 cells are another subset of pro-inflammatory regulatory T cells defined by their production of interleukin 17 (IL-17). In a variety of types of cancer, γδ T17 cells promote the accumulation and expansion of immunosuppressive cells and expedite the development of cancer [16].

## Plasticity of γδ T cells

Interestingly, in response to different cytokines, γδ T cells can shift from one phenotype to another, in a process referred to as polarization [36, 37]. This property is referred to as the plasticity of γδ T cells. It has been reported that Vγ9 Vδ2 T cells can be polarized into γδ T17 cells (producing only IL-17), γδ T1/17 cells (producing both IFN-γ and IL-17), γδ T1 cells (producing both IFN-γ and TNF-α) and γδ T2 cells (producing increased IL-4) with distinct cytokines being required for their polarization initiation and maintenance [38]. In this regard, it has been shown that IL-6, IL-1β, and TGF-β are required to generate γδ T17 cells in neonates, whereas γδ T1/17 cells additionally require IL-23. In adults, memory γδ T1/17 cells and γδ T17 cells, required IL-23, IL-1β, and TGF-β but not IL-6 [39]. For γδ T1 cell and γδ T2 cell polarization, Vγ9 Vδ2 γδ T cells should be stimulated with isopentenyl pyrophosphate (IPP) in the presence of Th1-priming (IL-12, anti-IL-4 Ab) or Th2-priming (IL-4, anti-IL-12 Ab) conditions [40]. It has also been shown that Vγ9 Vδ2 γδ T cells can polarize toward FOXP3+ γδ Treg cells following stimulation with TGF-β and IL-15 in vitro [41]. Moreover, γδ T cells can polarize towards follicular B-helper T cells (γδ Tfh cells) following stimulation with IPP and IL-21, which facilitate maturing B cells to produce antibodies against foreign antigens. All in all, this broad polarization range indicates that there are no clear boundaries between the structural and functional subsets of γδ cells, and it is possible to polarize Vδ2 T cells into nearly all functional subsets with distinct cytokine stimulation. Cytokines, by determining γδ T cell polarization, therefore ultimately define the role of γδ T cells in cancer.

Because of plasticity of γδ T cells, these cells can therefore be viewed both as being a “friend” or a “foe” of cancer. As a “foe” of cancer, γδ T cells exert both direct and indirect antitumor effects, principally due to Vγ9 Vδ2 T cells, γδ Tfh cells, γδ T1 cells, as well as Vδ1 γδ T cells. The term “friend of cancer,” refers to a subset of γδ T cells, including γδ T17 cells, γδ Treg cells, and Vδ1 γδ T cells. It should be noted that the role of Vδ1 γδ T cells in cancer is controversial since they have been suggested to have both an antitumor role and a protumor role [14, 42–44].

## γδ T cells as foes in cancer development

### Specific γδ T cells subsets play a direct antitumor role

**Fig. 2:**
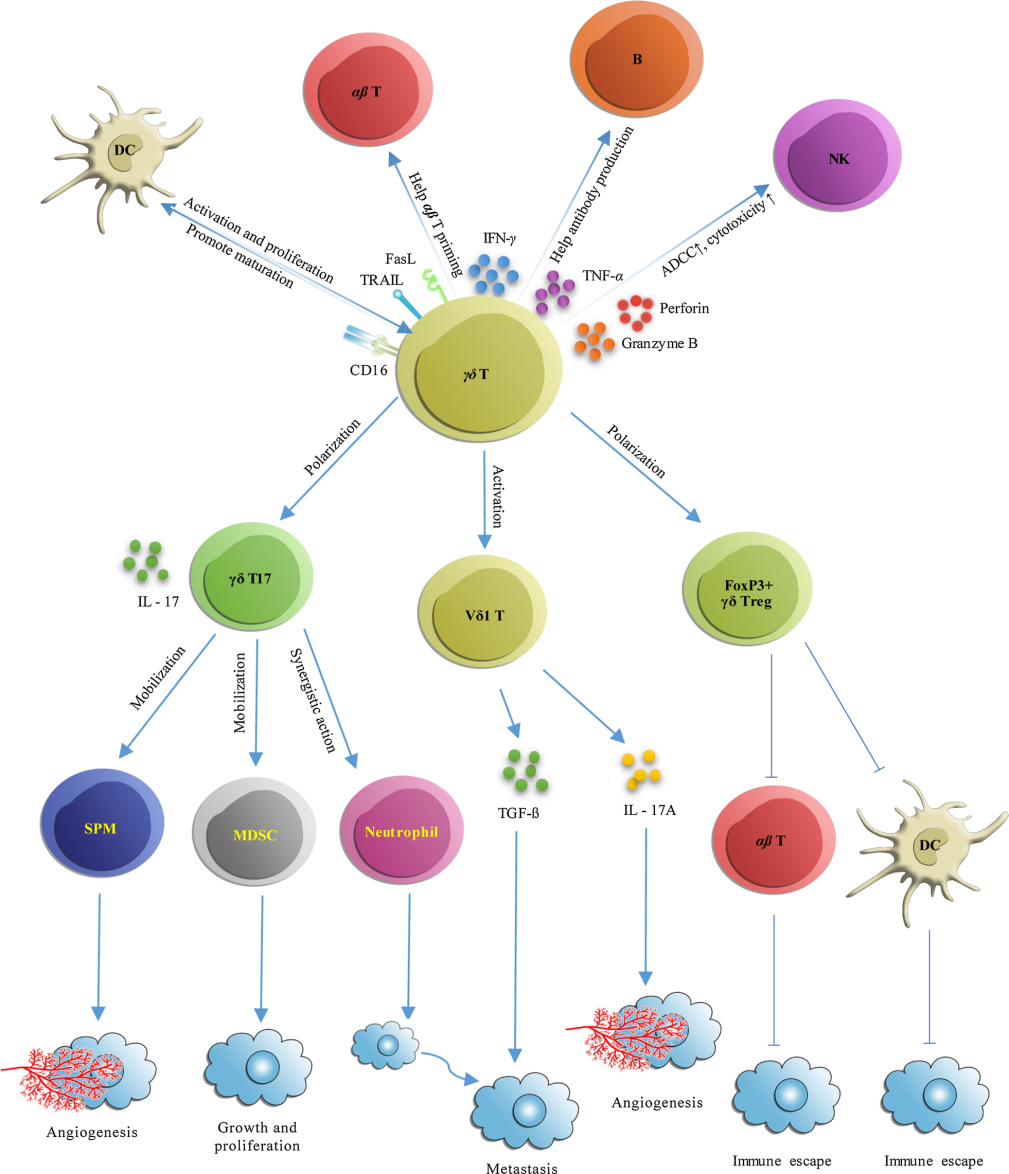
Antitumor and protumor functions of γδ T cells. γδ T cells have both direct and indirect antitumor effects. Direct antitumor effects are mediated by lysing the tumor through the perforin-granzyme pathway, providing an early source of the inflammatory cytokines such as IFN-γ and TNF-α, eliminating Fas+ and TRAIL-R+ tumor cells, and ADCC. The indirect antitumor role of γδ T cells is mediated by polarized γδ Tfh cells, which promote B-cell antibody secretion. Besides, γδ T cells also present antigens for αβ T cell priming, trigger dendritic cell (DC) maturation, and induce robust NK cell-mediated antitumor cytotoxicity to play indirect antitumor role. With regard to their protumor effect, γδ T cells can polarize into FOXP3+ γδ Treg cells, and γδ T17 cells. In addition, Vδ1 T cells are another subset of γδ T cells that possess protumor activity. γδ T cells are able to directly impair αβ T cells and DC antitumor immunocyte function. γδ T cells can also enhance MDSC, SPM, and neutrophil immunosuppressive functions. Together, these actions promote tumor angiogenesis, growth, proliferation, metastasis, and immune escape

The direct antitumor role of γδ T cells has been documented from four different aspects (Fig. 2). First, after migrating to the tumor local environment, γδ T cells can lyse cancer cells through the perforin-granzyme pathway [45]. For example, inhibiting the perforin-granzyme secretion capacity of Vγ9 Vδ2 T cells reduces their ability to lyse breast cancer cell in vitro [46]. In renal cancer, γδ T cells display a selective lytic potential toward autologous primary renal cancer cells, but not against normal renal cells, mainly depending on the TCR and the NKG2D receptor. This lytic activity also involves the perforin-granzyme pathway [47]. In an in vitro study of head and neck squamous carcinomas, perforin-granzyme lytic activity was also derived from γδ T cells [48].

Second, γδ T cells can eliminate cancer cells through the ligands TRAIL and FasL [49, 50]. If TRAIL is blocked, γδ T cell-mediated cytotoxicity activity is reduced [51]. Upregulation of Fas on osteosarcoma cells reportedly results in an enhanced susceptibility of the cells to γδ T cell lysis [52].

Third, γδ T cells can kill cancer cells via ADCC. ADCC occurs when CD16 (FcγR III) present on γδ T cells. CD16 binds to the Fc region of IgGs, which constitutes another means of γδ T cell target recognition, in addition to TRAIL and FasL. CD16 can also be up-regulated on γδ T cells, depending on the precise biological situation. Binding to its target may trigger either cytotoxicity or other effector functions (e.g., IFN-γ secretion) [53, 54]. The existence of the γδ T cell effect was also proven by Zheng et al. who cloned the extracellular domains of a Vγ9 Vδ2 TCR from ovarian cancer TILs, and conjugated them with Fc domain of human IgG1 [55]. This chimeric antibody mediated cell killing via ADCC in a dose-dependent manner. In vivo, this TCR Vγ9 Vδ2 (OT3)-Fc significantly inhibited cancer cell growth and enhanced survival in human ovarian carcinoma xenograft models.

Lastly, γδ T cells are important early sources of IFN-γ and TNF-α [56, 57]. Both IFN-γ and TNF-α inhibit cancer growth through several mechanisms, including the enhancement of antitumor immunity, and the inhibition of cancer angiogenesis [58]. The secretion of IFN-γ and TNF-α by γδ T cells is promoted by numerous stimuli, including TCR agonists, ligands of NKG2D, and certain cytokines, such as IL-12 and IL-18 [59].

### Specific γδ T cells subsets exert an indirect antitumor effect

γδ T cells exert their indirect antitumor effect by interacting with B cells, DCs, αβ T cells, and NK cells, respectively (Fig. 2). γδ T cells have been shown to affect B cell antibody secretion in non-immunized mice. Huang et al. have shown that selective ablation of Vγ4 and Vγ6 γδ subsets of γδ T cells, rather than removing all of the γδ subsets, strongly affect serum Ab levels in non-immunized mice. This demonstrates that γδ T cells are capable of modulating the population of pre-immune peripheral B cells and their antibody productivity [60]. Caccamo et al. have observed that after co-culture of Vγ9 Vδ2 T cells with IPP and IL-21, the Vγ9 Vδ2 T cells polarized toward a lymphocyte subset displaying features of follicular B-helper T (Tfh) cells. These Tfh-like Vγ9 Vδ2 T cells could secrete IL-4, IL-10, and CXCL13, and help B cells to produce antibody in vitro. These results are in line with Bansal et al. who demonstrated that Vγ9 Vδ2 T cells express Tfh cells markers when stimulated with IL-21 and HMB-PP, indicating that they are able to help B cells to produce antibodies, just like Tfh cells [61, 62].

γδ T cells can also act as antigen presenting cells (APCs) for αβ T cell priming. As far back as 2006, γδ T cells were first reported to have an antigen presenting function. After stimulation, the expression levels of antigen presenting molecules in γδ T cells increased, including the levels of leukocyte activation receptor (CD69), the antigen presenting molecule (HLA-DR), and T cell co-stimulation and adhesion molecules (CD80, CD86, CD54, and CD40) [63]. In contrast to αβ T cells, γδ T cells, as antigen presenting cells, are able to up-regulate CD36, a scavenger receptor involved in the uptake of apoptotic cells by immature DCs and macrophages. Because of a high level of expression of CD36, γδ T cells kill liver cancer cells, followed by uptake of their debris, and through their APC function induce a cancer antigen-specific CD8+ T cell response [64]. Brandes et al. found that activated Vγ9 Vδ2 T cells induce proliferation of naïve CD4+ αβ T cells, and promote their differentiation into cytotoxic T lymphocytes (CTLs) [65].

γδ T cells can also trigger DC maturation. In return, DCs can also induce the activation and proliferation of γδ T cells, enhancing their cytotoxic and immunoregulatory functions [66], which demonstrate that each of these cell types can act individually to remove cancer cells, but they can also interact synergistically [67]. Devilder et al. found that Vγ9 Vδ2 T cells can accelerate the maturation of DCs. This DC maturation relies on a combination of cytokine TNF-α and cell contact dependent signals [68]. In another study, Conti et al. also point out that DC activation by γδ T cells was almost all mediated through TNF-α and IFN-γ, and the authors further noted that this activation required CD86 and cell to cell contact (i.e. between DCs and γδ T cells) [69]. In return, immature DCs, and to a lesser extent mature dendritic cells (mDCs), are capable of enhancing the ability of Vγ9 Vδ2 T cells to secrete TNF-α [70].

Finally, γδ T cells can induce robust NK cell-mediated antitumor cytotoxicity through CD137 engagement. Maniar et al. have shown that in vitro expanded γδ T cells can enhance NK cell cytotoxicity to NK-resistant cancers [71]. This enhanced NK cell cytolysis requires immobilized human IgG1, and co-stimulation between CD137 expressed on NK cells and CD137L expressed on γδ T cells.

## γδ T cells as friends in cancer development

### Specific γδ T cells subsets promote cancer progression directly

γδ T17 cells have been reported to support cancer progression by promoting angiogenesis in gallbladder cancer, ovarian cancer, as well as others [11, 72]. γδ T17 cells are the major source of IL-17, which plays an immunosuppressive role in cancer. In gallbladder cancer (GBC), γδ T17 cells migrate toward the tumor bed through the CXCL9-CXCR3 axis. IL-17, secreted by γδ T17 cells, then induces the production of vascular endothelial growth factor, as well as other angiogenesis related factors. The presence of γδ T17 cells has been associated with poor survival in GBC patients [11]. After exposure to IL-6 and TGF-β, tumor-infiltrating CCR6(−) γδ T cells can be polarized toward γδ T17 cells. Furthermore, IL-17-deficient mice showed markedly reduced angiogenesis and consequently slower cancer progression, suggesting a significant role for γδ T17 cells in cancer cell growth (Fig. 2).

It has been well demonstrated that Vγ9 Vδ2 γδ T cells can polarize toward FOXP3+ γδ Treg cells following stimulation with TGF-β and IL-15 in vitro [41]. These FOXP3+ γδ Treg cells have a similar function as αβ Treg cells, which suppress the proliferation of anti-CD3/anti-CD28 stimulated PBMCs. Additionally, Vδ1 Treg cells have also been found to be induced in an immune suppressive TME. In breast cancer, Vδ1 Treg cells, attracted by breast cancer cells, have also been shown to secrete the chemokine IP-10 [35].

Vδ1 γδ T cells are another subgroup of recently discovered γδ T cells with protumor activity. Vδ1 γδ T cells are involved in inflammation-induced cancer progression, dependent on the production of IL-17A [73]. In another report, Vδ1 γδ T cells have been reported to strongly secrete TGF-β. The secreted TGF-β can induce the epithelial to mesenchymal transition during which the cancer can escape immune detection, ultimately resulting in metastasis and cancer invasiveness [74]. With regards to T helper cell suppression, peripheral human Vδ1 γδ T cells have a more potent regulatory potential than αβ Treg cells (CD4+ CD25+ cells) [66, 75]. Therefore, Vδ1 γδ T cells are able to modulate the immune system, the TME, and promote cancer cell invasiveness and metastasis.

In addition, a Vδ1 and Vδ2 γδ T cell imbalance (i.e. an increase in the Vδ1:Vδ2-ratio) has also been proven to contribute to the development of cancer [76–78]. This Vδ1 and Vδ2 γδ T cell imbalance mediated by IL-4. IL-4 inhibits the activation of naïve Vδ1 γδ T cells, in a TCR-STAT6 dependent manner, and in doing so, promotes the growth of activated Vδ1 γδ T cells and subsequently up-regulates the number of Vδ1 γδ T cells. These Vδ1 γδ T cells secrete IL-10 resulting in the inhibition of Vδ2 γδ T cells [79]. In the presence of IL-4, Vδ1 γδ T cells secrete significantly less IFN-γ, more IL-10, and express lower NKG2D, compared with Vδ2 γδ T cells.

### γδ T cells impair the function of other antitumor immunocytes

As a “friend” of cancer, γδ T cells can impair the antitumor ability of immunocytes. For example, it has been reported that human breast tumor infiltrating Vδ1 γδ T cells could inhibit DC maturation and their APC functions, thus impairing naïve αβT cell activation and differentiation into effector T cells (CD4+ and CD8+ T cells) through the TLR8 signaling pathway [44]. In pancreatic ductal adenocarcinoma, it has been shown that γδ T cells express high levels of PD-L1 and support pancreatic oncogenesis by restraining αβ T cell activation [80]. It has also been discovered that tumor-derived γδ Treg cells can induce cell cycle arrest of responder T cells, and that they can suppress naïve and effector T cells through the induction of T cell senescence. Further, γδ Treg cells have been shown to induce DC senescence, resulting in an impairment of their phenotypic and functional features [81].

### γδ T cells enhance immunosuppressive cell function

MDSCs have been reported to facilitate cancer progression in several types of cancers, including: breast cancer, colorectal cancer (CRC), and pre-hepatic carcinoma [15, 16, 82, 83]. IL-17 is one of the main chemo-attractant driving forces for the recruitment of MDSCs [16, 82, 83]. In one CRC study, innate γδ T17 cells could convert cancer-elicited inflammation into immunosuppression through MDSCs; furthermore, cancer γδ T17 cells were correlated with clinicopathological features of human CRC [16]. Research has also demonstrated that γδ T cells play a regulatory role in immune tolerance by mobilizing MDSC infiltration to the liver, leading to MDSC-mediated CD8+ T cell exhaustion [84]. γδ T cells secreting dermal IL-17 play a critical role in skin inflammation, and this inflammation is capable of inducing MDSCs that facilitate cancer progression by counter-acting immune surveillance and allowing for the outgrowth and proliferation of malignant cells [85, 86]. In a mouse ovarian cancer model, IL-17 secreted by CD27(−) Vγ6(+) γδ T cells was found to immobilize small peritoneal macrophages (SPMs). These SPMs up-regulate protumor and pro-angiogenic molecular mediators, and induce ovarian cancer growth [87].

In addition, γδ T cells are capable of affecting the function of neutrophils in breast cancer. Research has shown that IL-1β and IL-17 secreted by γδ T17 cells stimulate the expansion and polarization of neutrophils. These tumor-induced neutrophils acquire the ability to suppress cytotoxic T lymphocytes carrying the CD8+ antigen, which in turn facilitates the establishment of metastases [88]. In addition, these neutrophils have been found to be able to suppress peripheral Vγ9 Vδ2 T cells [89, 90]. In this way, neutrophils impair antitumor Vγ9 Vδ2 T cells, while at the same time the synergy with γδ T17 cells create an immunosuppressive TME.

Taken together, γδ T cells can enhance the accumulation and function of immunosuppressive cells. These immunosuppressive cells can convert tumor-elicited inflammation into immunosuppression and promote cancer angiogenesis.

## Clinical application of γδ T cells

Following a review of the γδ T cell clinical trials conducted over the last decade, either through adoptive transfer or in vivo expansion, it is clear that γδ T cell therapy is safe (Table 2). γδ T cell based cancer immunotherapy can be divided into two categories based on either activation or expansion. The basis of the first method is to stimulate γδ T cells in vivo by systemic administration of phosphoantigens or nitrogen-containing bisphosphonates (N-bis). The basis of the second method is to expand γδ T cells sourced from peripheral blood mononuclear cells (PBMCs) ex vivo using synthetic phosphoantigens or N-bis, followed by administration of the cultured γδ T cells to the patient. γδ T cells based immunotherapy have been applied to variety types of solid cancer and hematological malignancies, in which the most wide of usage is in renal cell carcinoma therapy.

**Table 2:** Clinical trials using γδ T cell based cancer immunotherapies

Cell types	Diseases	Cell sources	Number of patients	Phase of clinical trail	Outcome			References
					Response (n)	RR (%)	CBR (%)	
Vγ9 Vδ2 T	Prostate cancer	PBMC	18	I	SD:5; PR:3; PD:3; NE:7	27	73	Dieli et al. [50]
γδ T	NSCLC	PBMC	10	I	SD:3; PD:5; NE:2	0	38	Nakajima et al. [91]
Vγ9 Vδ2 T	NSCLC	PBMC	15	I	SD:6; PD:6; NE:3	0	50	Sakamoto et al. [92]
LAK:αβT, γδT, NK	Breast cancer	PBMC	20	I	PR:3; SD:1; PD:6; NE:10	30	40	Noguchi et al. [93]
Vγ9 Vδ2 T	Lung cancer, stomach cancer, others	PBMC	5	I	PD:2; SD:2; NE:1	0	50	Noguchi et al. [93]
Vγ9 Vδ2 T	RCC	PBMC	12	I	SD:7; PD:1; NE:4	0	88	Lang et al. [94]
γδ T	RCC	PBMC	11	I/II	CR:1; SD:5; PD:5	9	55	Kobayashi et al. [95]
Vγ9Vδ2 T	CRC	PBMC	6	I	CR:1; PR:4; NE:1	100	100	Izumi et al. [96]
γδ T	NSCLC	PBMC	15	I	SD:6; PD:6; NE:3	0	50	Kakimi et al. [97]
Vγ9Vδ2 T	RCC	In vivo expansion	10	I	PR:1; SD:6; NE:3	14	100	Bennouna et al. [98]
LAK:αβT, γδT, NK	RCC, MM, AML	In vivo expansion	21	I/II	CR:6; PR:2; PD:12; NE:1	40	40	Kunzmann et al. [103]
LAK:αβT, γδT, NK	Advanced hematological malignancies	In vivo expansion	4	I	CR:3; PD:1	75	75	Wilhelm et al. [99]
Total			147		SD:41; CR:11; PR:13; PD:47; NE:33	21	57	

*PBMC* peripheral blood mononuclear cell, *LAK* lymphokine activated killer cell, *NSCLC* non-small cell lung carcinoma, *RCC* renal cell carcinoma, *MM* multiple myeloma, *AML* acute myelocytic leukemia, *CR* complete response, *PR* partial response, *SD* stable disease, *PD* progressive disease, *NE* not evaluable, *RR* response rate, RR = (CR + PR)/number of evaluable patients, *CBR* clinical benefit rate, CBR = (CR + PR + SD)/number of evaluable patients, *CRC* colorectal cancer

Pioneering trials have defined conditions for the safe use of phosphoantigens and zoledronate for the activation of γδ T cells in patients. The most common side effect flu-like symptoms without γδ T cell expansion is generally induced with low doses of stimuli. Most of the adverse effects are in grade 1–2: fever, fatigue, elevation of liver transaminase, and eosinophilia [90]. Grade 3 and 4 severities of adverse events that have recurrently been reported are characterized by thrombophlebitis, thrombosis, hyperglycemia, hypocalcemia, chest and musculoskeletal pain, gastritis, myocardial infarction and renal toxicity [100].

While the safety of γδ T cell activation in patients has been proven, and the pharmacodynamics of phosphoantigens administered to humans has been established, the issue of limited efficacy still remains with an average response ratio of only 21% and an average clinical benefit rate of only 57%. This problem could be related to activation-induced γδ T cell anergy, as well as to a decrease in the number of peripheral blood γδ T cells after infusion of the stimulants. All of these phenomena may arise as a result of properties of γδ T cells [100, 101].

The γδ T cell anergy and the decrease in the number of peripheral blood γδ T cells after infusion are qualitative and quantitative problems in γδ T cell therapy. With regard to the qualitative problem, the cytotoxicity of γδ T cells may be affected by the suppressive TME as well as the cancer stage, both of which could limit the antitumor function of γδ T cells. In pancreatic carcinoma, γδ T cell cytotoxicity ability was diminished by the levels of soluble MICA/B in the TME [101]. With regard to the quantitative problem, it is rational to believe that γδ T cell polarization may result in a decrease in the number of antitumor γδ T cells, where cytokines such as IL-23, IL-15, and TGF-β largely influence cell polarization. In skin squamous cell cancer (SCC), significantly more γδ T17 cells were found in SCC patients with advanced disease (stages III and IV), compared to patients with early disease (stages I and II). In contrast, the frequencies of Vδ2 γδ T cells were higher in SCC patients at stages I and II, but significantly decreased in patients with advanced disease (stages III and IV) [102]. This antitumor and protumor γδ T cell composition shift sheds light on the possibility that γδ T cell polarization limits their immunotherapy efficiency. Both anergy and polarization of γδ T cells result in a reduction in their antitumor activity. Appropriate methods are therefore needed to modulate this to allow us to benefit from γδ T cell immunotherapy in the long run.

To a large extent, host immune status may also affect γδ T cell adoptive immunotherapy, but only a few clinical trials have evaluated immune status before γδ T cell adoptive immunotherapy. Host immune status arises from a number of different aspects, such as composition of the TME cells, the action of immune checkpoints, cytokine levels and so on. In the context of γδ T cell adoptive immunotherapy, immune checkpoints such as Programmed cell death protein 1 (PD-1), potent immunosuppressive cytokines such as IL-17 and IL-4, and relevant immunocytes such as neutrophils are all involved in γδ T cell cytotoxic immune responses. Therefore, an evaluation of the patient’s immune status before commencing γδ T cell immunotherapy will minimize the likelihood of treatment failure and provide the patient with the most appropriate treatment options.

To broaden the application of γδ T cell-based adaptive immunotherapy, there are still many limitations that need to be resolved, such as how to switch the suppressive TME into a normal environment, and how to attract more γδ T cells capable of targeting cancer cells.

## Prospects for γδ T cell-based immunotherapies

**Fig. 3:**
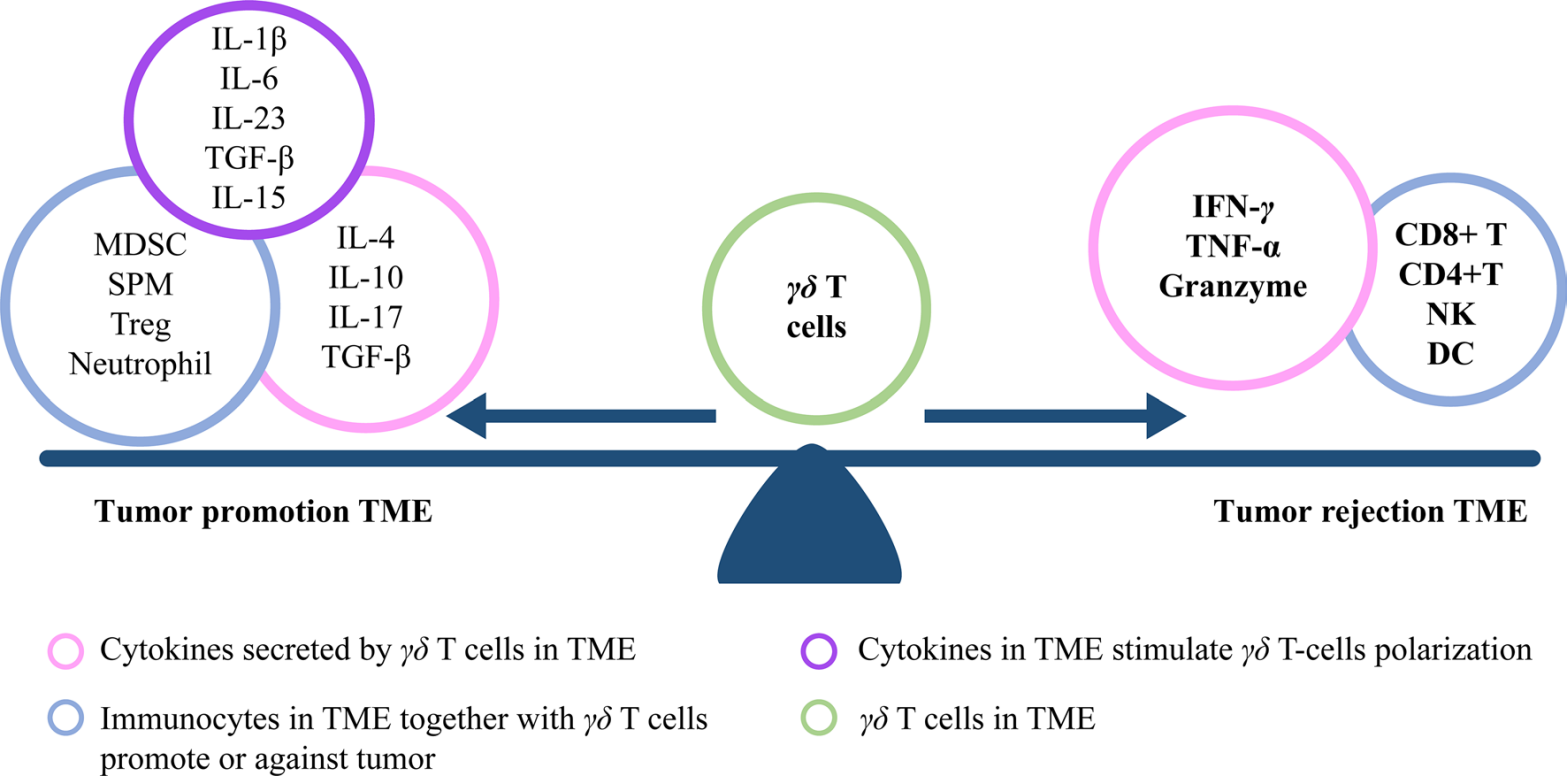
The functions of γδ T cells are influenced by the TME. The balance of pro- and anti-inflammatory cytokines and the cellular state govern the function of γδ T cells. Tumor and stromal cells produce a variety of cytokines and chemokines that either contribute to or disrupt the development of a pro-tumorigenic niche. These factors can also reprogram γδ T cells to adopt a protumor or antitumor state

γδ T cell-based immunotherapies are imperfect, which likely arises from the fact that only certain of the γδ T cells have robust antitumor effects, whereas others have a potent protumor function. These aspects will be discussed below from the viewpoint of the cytokine effects and the suppressive TME (Fig. 3) [103].

As either “foe” or “friend” of cancer, γδ T cells are double-faced immunocytes that play a role in cancer progression. As discussed above, γδ T cells are a group of heterogeneous T cells, the combinatorial variety generated by the different TCRs are thought to explain why γδ T cells exert diverse actions in distinct TME, such as Vδ1 γδ T cells, have a controversial role in cancer immunity. It is logical to conduct γδ T cell functional identification and elimination protumor subgroup before the γδ T cells are transferred into the patient. Furthermore, in vivo expansion creates a possibility for protumor subset proliferation to occur. This indicates that in vivo expansion should avoid an immunosuppressive TME, which increases the possibility of γδ T cells protumor polarization. To solve this problem, we suggest that γδ T cell-based immunotherapies should be conducted at an early TNM stage to avoid an immunosuppressive TME, and that combination of γδ T cell-based immunotherapies with chemotherapy or multi-immunocyte immunotherapy could also be used against the stubborn immunosuppressive TME. Combination of γδ T cells with other cytotoxic T cells [e.g., αβ T cells or cytokine-induced killer (CIK) cells] might also enhance therapeutic efficacy, owing to a two-pronged synergism: non-MHC restricted γδ T cells and tumor-specific adaptive response T cells. In so doing, multi-immunocyte adoptive immunotherapy will widen the scope of immune responsiveness to include cancer cells, and even cancer stem cells [104, 105].

Moreover, γδ T cells are capable of changing their function in response to cytokine stimulation. Depending on the stimulation by different cytokines in TME, they can change their function toward being either antitumor or protumor. Therefore, cytokine balance is a very important factor in the tumor immune microenvironment. Artificial modification of the TME cytokine balance could be another promising way to amplify the effect of γδ T cells. In this regard, the important cytokines include IL-23, IL-1β, IL-15, IL-17, IL-4, IL-10, IL-36γ, and TGF-β. We advocate that levels of IL-17 should be routinely measured to predict the immune status before γδ T cell adoptive immunotherapy is initiated. There are also some cytokines in the TME that enhance the antitumor function of γδ T cells and inhibit the protumor γδ T cells responses. As IL-21 has been proven capable of inhibiting γδ T 17 cell protumor cell responses, further research on IL-21 should be conducted in human cancer [106]. It has been shown that IL-36γ acts synergistically with TCR signaling and/or that IL-12 can stimulate γδ T cells. IL-36γ is able to promote IFN-γ production by CD8+ T cells, NK cells, and γδ T cells. IL-36γ transforms the TME in favor of cancer eradication and exerts strong antitumor effects [107]. IL-18 has also been found to have the ability to promote the expansion of γδ T cells with potent antitumor activity, such as those that produce GM-CSF, IFN-γ, and TNF-α at high levels [108, 109]. Furthermore, the addition of IL-15 to γδ T cell cultures also results in a more activated phenotype, a higher proliferative capacity, a more pronounced T helper 1 polarization, and an increased cytotoxic capacity of γδ T cells [110]. All of these data support the rationale of exploring the use of cytokines in clinical adoptive therapy protocols that employ γδ T cells.

Combination of γδ T cells adoptive immunotherapy with immune check point inhibitors is another approach that could be used to enhance the antitumor activity of immune effector of these cells. As the above research has shown, the interaction between PD-1 on αβ T cells and its ligand PD-L1 on γδ T cells restrains αβ T cell activation. Logically, therefore another tactic is to block the immune checkpoint interaction in order to enhance the cytotoxic activity of antitumor cells. In this regard, and beyond PD-1, there are many immune checkpoint inhibitors that target molecules such as CTLA-4, IDO, VISTA, Galectin-9, LAG-3, and TIM-3. Moreover, ten cell surface proteins have been identified that were statistically differentially expressed between “γδ-susceptible” and “γδ-resistant” hematopoietic malignancy. Three of these genes (ULBP1, TFR2, and IFITM1) are associated with increased susceptibility to Vγ9 Vδ2 T cell cytotoxicity, whereas the other seven (CLEC2D, NRP2, SELL, PKD2, KCNK12, ITGA6 and SLAMF1) are enriched in resistant cancers. These immune checkpoints therefore provide a wide range of different ways to improve γδ T cell adoptive immunotherapy [102–116].

## Conclusions

The limited efficiency of γδ T cell based cancer immunotherapy may be because of their dual nature. Their actions as either “friends” or “foe” of cancer is heavily influenced by the cytokines present in the TME. A mixture of both anti- or pro-tumor γδ T cells used in adoptive immunotherapy, coupled with the fact that γδ T cells can be polarized from antitumor cells to protumor cells are likely reasons for the low efficacy seen with γδ T cells. This review is the first to analyze the dual effect mechanism of γδ T cells and we further propose a means to improve the effect of γδ T cells. The future holds the promise of depleting the specific protumor γδ T cell subgroup before therapy, multi-immunocyte adoptive therapy, modification of the cytokine balance in the TME, and the combination of γδ T cells adoptive immunotherapy with immune checkpoint inhibitors. Only if we properly handle these cells, can we benefit from γδ T cell immunotherapy in the long run.

## Abbreviations

AML: acute myelocytic leukemia
ADCC: antibody-dependent cellular cytotoxicity
APC: antigen presenting cells
CAR: chimeric antigen receptor
CR: complete response
CRC: colorectal cancer
CBR: clinical benefit rate
CTLs: cytotoxic T lymphocytes
CTLA-4: cytotoxic T lymphocyte-associated protein-4
DCs: dendritic cells
GBC: gallbladder cancer
IL-17: interleukin-17
LAK: lymphokine activated killer cells
mDCs: myeloid dendritic cells
MSC: mesenchymal stem cell
MDSC: myeloid derived suppressor cells
MM: multiple myeloma
MICA: MHC class I chain-related molecule A
MICB: MHC class I chain-related molecule B
NE: not evaluable
NSCLC: non-small cell lung carcinoma
PBMC: peripheral blood mononuclear cells
RCC: renal cell carcinoma
RR: response rate
PD: progressive disease
PD-1: programmed death-1
PR: partial response
SCC: squamous cell cancer
SD: stable disease
SPMs: small peritoneal macrophages
TCR: T cell receptor
Tfh: follicular B-helper T
TME: tumor microenvironment
TRAIL: tumor necrosis factor-related apoptosis-inducing ligand
