# Correction to: Gamma-delta (γδ) T cells: friend or foe in cancer development?

**DOI:** 10.1186/s12967-018-1491-x

**Published:** 2018-05-08

**Authors:** Yijing Zhao, Chao Niu, Jiuwei Cui

**Affiliations:** Cancer Center, The First Hospital of Jilin University, Changchun, 130021 People’s Republic of China

## Correction to: J Transl Med (2018) 16:3 10.1186/s12967-017-1378-2

Following publication of the original article [1], the authors reported that they omitted to state that parts of Fig. 2 were adapted from Van Acker et al. [2] published by Taylor & Francis Ltd (www.tandfonline.com). The authors apologise for this omission. Figure 2 and its corrected caption are given below.

**Fig. 2:**
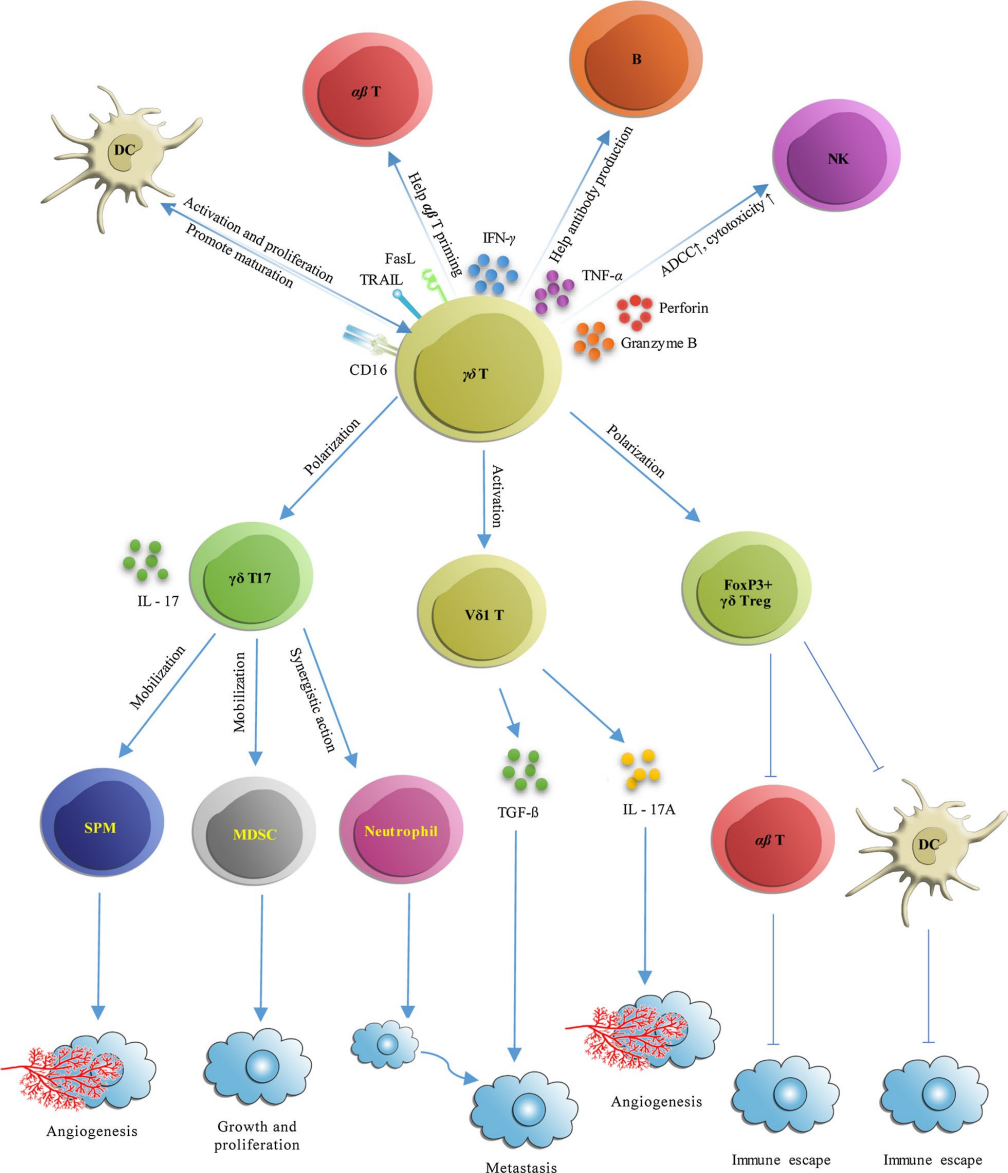
Antitumor and protumor functions of γδ T cells. γδ T cells have both direct and indirect antitumor effects. Direct antitumor effects are mediated by lysing the tumor through the perforin-granzyme pathway, providing an early source of the inflammatory cytokines such as IFN-γ and TNF-α, eliminating Fas+ and TRAIL-R+ tumor cells, and ADCC. The indirect antitumor role of γδ T cells is mediated by polarized γδ Tfh cells, which promote B-cell antibody secretion. Besides, γδ T cells also present antigens for αβ T cell priming, trigger dendritic cell (DC) maturation, and induce robust NK cell-mediated antitumor cytotoxicity to play indirect antitumor role. With regard to their protumor effect, γδ T cells can polarize into FOXP3+ γδ Treg cells, and γδ T17 cells. In addition, Vδ1 T cells are another subset of γδ T cells that possess protumor activity. γδ T cells are able to directly impair αβ T cells and DC antitumor immunocyte function. γδ T cells can also enhance MDSC, SPM, and neutrophil immunosuppressive functions. Together, these actions promote tumor angiogenesis, growth, proliferation, metastasis, and immune escape (Parts of this figure are adapted from Van Acker et al.)
